# Nine (not so simple) steps: a practical guide to using machine learning in microbial ecology

**DOI:** 10.1128/mbio.02050-23

**Published:** 2023-12-21

**Authors:** Corinne Walsh, Elías Stallard-Olivera, Noah Fierer

**Affiliations:** 1 Cooperative Institute of Research in Environmental Sciences, CU Boulder, Boulder, Colorado, USA; 2 Ecology and Evolutionary Biology Department, CU Boulder, Boulder, Colorado, USA; Instituto Carlos Chagas, Curitiba, Brazil

**Keywords:** machine learning, microbial ecology, predictive modeling, microbiome

## Abstract

Due to the complex nature of microbiome data, the field of microbial ecology has many current and potential uses for machine learning (ML) modeling. With the increased use of predictive ML models across many disciplines, including microbial ecology, there is extensive published information on the specific ML algorithms available and how those algorithms have been applied. Thus, our goal is not to summarize the breadth of ML models available or compare their performances. Rather, our goal is to provide more concrete and actionable information to guide microbial ecologists in how to select, run, and interpret ML algorithms to predict the taxa or genes associated with particular sample categories or environmental gradients of interest. Such microbial data often have unique characteristics that require careful consideration of how to apply ML models and how to interpret the associated results. This review is intended for practicing microbial ecologists who may be unfamiliar with some of the intricacies of ML models. We provide examples and discuss common opportunities and pitfalls specific to applying ML models to the types of data sets most frequently collected by microbial ecologists.

## INTRODUCTION

Microbial ecologists are increasingly turning to machine learning (ML) models to better identify and predict meaningful biological patterns and relationships in molecular microbial community data (including data generated via marker gene or metagenomic sequencing). Whether one is studying the microbiomes associated with animal or plant hosts, or the microbiomes found in terrestrial, aquatic, or engineered systems, the microbial communities are typically complex with a broad diversity of taxa and genes represented in a given sample, a high degree of variability across samples, and complex associations among organisms within each sample. ML can help overcome some of these challenges by requiring few *a priori* assumptions about the system, being robust to non-normal data, and incorporating the complexity of biological communities. While ML models are typically associated with prediction at the expense of understanding, they can also offer opportunities to extract interpretable information from unwieldly microbiome data. To cite a few (of many) recent examples of ML applications in microbiome studies, such models have been used to predict crop productivity using bulk soil metagenomic information (1), identify nuclear waste and oil spill contamination in wells and ocean water using microbial taxonomic information (2), determine the environmental optima for bacterial growth based on a subset of genes (3), and identify or predict human disease states from shifts in human host microbiome composition (4, 5).

There are many resources available for biologists who want to use machine learning (6–9), but microbial ecology data have unique features that require specific considerations. Here, we offer a step-by-step guide to using ML with the specific data types commonly generated by microbial ecologists and the questions they are often trying to answer with those data. We note that there are other resources for microbial ecologists that comprehensively detail the many types of ML models or review examples of their applications (10–15). Our goal with this piece is to provide a more practical guide for developing ML models from input data to interpretation. We focus here on the applications of supervised ML models (when data are labeled, see Table 1) to analyze data on the taxonomic composition of microbiomes or their functional gene profiles obtained via high-throughput sequencing of microbial DNA. We recognize that these are not the only types of data collected by microbial ecologists, or the only model types available, but together they represent the most common scenarios in the field of microbial ecology. Use of unsupervised machine learning approaches, such as neural networks and “deep” learning, also has applications in microbial ecology but is beyond the scope of this work. We discuss challenges of implementing supervised ML models that are commonly encountered when applying these methods to microbial ecology questions. Specifically, we focus on when it may be useful to construct supervised ML models with microbiome data, how to do so, and how to interpret the model results effectively.

**TABLE 1:** Glossary of terms

Accuracy	The ratio of correctly categorized instances over the total number of instances; optimize for accuracy when the costs of both false positives and false negatives are symmetrical.
(AU)ROC curve	A metric measuring a binary classifier’s ability to distinguish between positive and negative classes. More valuable as a metric when classes are balanced, and the cost of misclassification is consistent among classes. A value above 0.5 indicates that a model classifies more true positives and negatives than false positives and negatives.
(AU)PR/precision-recall curve	A metric measuring the tradeoff between precision and recall for a binary classifier. Superior to (AU)ROC when classes are not balanced (unequal number of samples in each class).
Bias	In machine learning, a model with high bias makes more simplifying assumptions about the data, potentially failing to take into account important information and underfitting. In other words, systematic error in the model results in inaccurate predictions.
Compositional data	Data in which variable values are only understandable relative to other variables, or ratios of a total value. For example, in taxonomic analyses via marker gene sequencing, abundances of identified taxa are typically presented in relative (proportional) terms and not absolute terms, meaning an increase in the number of reads in a sample does not necessarily mean that more of that organism was present.
Confounding (variables)	An extraneous variable that impacts both independent and dependent variables, potentially causing spurious associations within the data.
Cross-validation	A resampling procedure used to estimate and improve model performance. The model is trained and tested on different subsets of the data.
F1	The harmonic mean of precision and recall for a binary classifier. Because the F1 score gives equal weights to precision and recall, optimize for F1 score when seeking a balance between them and the cost of misclassification is consistent between classes.
Feature selection	Feature selection is the process of reducing the number of input variables in a data set by selecting only the most informative subset of variables.
Hinge-loss	Measures the probability that predicted outcomes for a binary support vector machine (SVM) match the observed outcomes; penalizes wrong and low confidence predictions more than log-loss.
Kappa	Takes into account the probability that correct classification can be achieved by random guessing, making it a more valuable metric than accuracy and F1 score for both unbalanced and multi-class data.
k-fold cross validation	A variant of cross-fold validation. With k-fold cross validation, the data set is split into k groups and for each group, one of the k groups is used as a test set and the remaining groups are used as the training set iteratively until each of the groups has been used as the test set. An extreme example of this is leave one out cross validation (LOOCV) where a single sample is iteratively selected for validation, eventually using the entire data set for validation.
Log-loss/cross-entropy loss	Measures the probability that predicted outcomes for a classification problem match observed outcomes.
MAE	Mean absolute error. Metric measuring the magnitude of errors between observed and predicted values for a regression problem. Does not give weight to larger errors as with (root) mean square error (RMSE), meaning outliers are not heavily penalized. Lower values are better.
MAPE	Mean absolute percentage error. Metric measuring the percentage difference between observed and predicted values for a regression problem, which can help with interpretability. However, MAPE values can be thrown off by sparse data and outliers, making it a poor choice for many applications in microbial ecology.
One-hot encoding	The process of turning non-ordinal categorical data into binary variables corresponding to each category.
Overfitting	When a model performs well on the training data set but poorly on the test data set.
Precision/positive predictive value	The number of true positives over the total number of predicted positives; optimize for precision when the cost of a false positive is high.
Predictor variables	Also known as “input data,” “features,” “explanatory variables,” “independent variables,” these are commonly microbial taxa or genes in microbial ecology data.
Response variables	Also known as a “dependent variable” or “outcome.” The variable that depends on variation in predictor variables and is the phenotype or value trying to be predicted.
Recall/sensitivity	The ratio of true positives over the total number of positive instances. Optimize for recall when the cost of a false negative is high. Also called the true positive rate (TPR).
(R)MSE	(Root) mean square error. Metric measuring the Euclidean distance between observed and predicted values for a regression problem, larger errors are penalized more heavily than smaller ones. Lower values are better.
“Sparse” data	Data where a large proportion of the values are zero. Most sequence-based data sets in microbial ecology are sparse.
Specificity	The ratio of predicted instances to true negative instances. Also called the true negative rate (TNR).
Supervised learning	A category of machine learning where input data are labeled, meaning there are both predictor values and response values associated with each sample.
Variance	In machine learning, a model with high variance makes fewer simplifying assumptions about the data, potentially taking too much noise into consideration and can lead to overfitting.
“Wide” data	Data where the total number of features is much larger than the total number of instances (e.g., more taxa or genes than samples).

### Step 1: Determine whether machine learning is necessary or even worth doing

Arguably, one of the most important questions to consider when determining whether to use ML algorithms is whether there is a hypothesized mechanism of interaction between the “input” data (microbial taxonomic or genetic information, also termed “predictors,” “features,” or “independent variables;” see Table 1) and the experimental question or response variable (also termed “output” or “dependent variable;” see Table 1). Classic statistical models (such as linear regression) allow for casual mechanistic inference and hypothesis testing but require explicit assumptions regarding how the data is structured, and *a priori* hypotheses of testable relationships within the data (16). In most biology research questions, simpler models and ease of inferences can be preferable to more complex models (if performance is similar) due to the importance of interpretability and generalizability. If the standard statistical approaches widely used by microbial ecologists (17, 18) are sufficient, there is no need to use ML models. More generally, machine learning might not be the best choice in contexts where the strengths of statistical modeling are particularly important, such as when there is a need for uncertainty estimates, non-automated data analysis, incorporation of prior knowledge, hypothesis testing, or addressing specific problems without the necessity for scalability or generalization. Machine learning models are typically employed when the more “standard” statistical approaches are not sufficient due to the structure of the data or when mechanistic inference is less important. However, it is important to note that machine learning cannot save bad study design or data: ML is not appropriate in situations where statistical methods would also struggle due to underlying issues like poor data quality, non-representative samples, or inherent biases in the data.

Machine learning models can detect associations in complex, non-normal data sets and enable prediction at some expense of mechanistic inference. Machine learning models do not require explicit assumptions about the underlying structure of the data and can identify non-linear relationships in the complex data sets typical of microbiome studies that are challenging to detect with more standard statistical approaches (16, 19). ML models especially excel when the goal is predicting an outcome, function, or phenotype from microbiome data. However, the results of ML models can be difficult to interpret in the context of biological understanding: you may be able to predict how big a plant will be based on the surrounding soil microbiome, but you may not necessarily be able to resolve how specific features of the microbiome independently contribute to observed differences in plant growth. That being said, there are some options for gaining biologically meaningful interpretations from ML output, as explained in later sections. In general, trying to predict a specific outcome in a complex system, such as diagnosing colorectal cancer with fecal microbiome data (20), is easier to address with ML approaches (21).

### Step 2: Get to know your data

The other critical question to ask when determining whether to use ML is “what do the data look like?” Taxonomic data or functional gene data that are commonly generated in microbiome studies require special handling whether statistical or ML modeling approaches are used (21). It is critical to assess how the issues detailed in Table 2 (compositionality, wideness/sparseness, uneven sampling depth, collinearities, confounders, garbage in garbage out [GIGO]; see Tables 1 and 2), or related data processing to address those issues, may affect downstream ML modeling efforts. In addition to considering the aforementioned issues, exploring the data visually via exploratory data analysis (EDA) is critical to do before attempting any ML modeling.

**TABLE 2:** Considerations for molecular microbial ecology data (not necessarily unique to ML)

Sequence data are often compositional (see Table 1)	DNA sequencing read counts are not independent or reflective of true abundance and may yield false correlations. Various transformation techniques such as log-ratio transformations and differential proportionality analysis can be applied to address compositional data (22–24). However, it is important to note that nonlinear machine learning algorithms are generally more robust to compositionality than linear approaches (25).
Sequence data often have uneven sampling depth (unequal read coverage) across samples	There is extensive discussion elsewhere on the options, pros, and cons of “normalizing” data to standardize read depths (26–28). Note that machine learning models can detect and propagate signals from sequencing depth if the input data are not normalized in some capacity. However, normalization procedures can also skew data in artificial ways (29). To mitigate this, performing model training on both transformed and untransformed data can identify major discrepancies due to data transformation. Additionally, microbiomes are rarely surveyed to completion (i.e., not all genes or taxa in a given sample are surveyed), so rare features may appear to be missing from some samples but not others. It is hard to confirm true absence, and thus, rare features should be treated with caution (see “GIGO”).
Sequence data are often wide and sparse	Sequence data are often wide—typically there are far more predictors (genes or taxa) than samples, and sparse—there are a lot of zero (null) values (e.g., the classic ecological principle of most species being rare [30]). This introduces specific types of error into ML models, most notably overfitting. Performing abundance/ubiquity-based filtering, grouping input variables into coarser groups (e.g., gene categories, functional taxonomic groups), and performing feature selection are all strategies to address the pitfalls of wide and sparse data.
Underlying relationships that introduce collinearities	Examples of collinearities include genes that correlate because they exist in the same taxon, or taxa that correlate because of geographic/physical proximity. Collinearity does not necessarily harm performance of ML models, but it can make ML models difficult to interpret as the underlying drivers can be obscured (31).
Confounders are signals that are not reflective of true biological variation related to the experimental question	“Batch effects,” such as reagents for DNA extractions, sequencing center, or day of data collection, can all introduce variation in microbial data that models could detect and propagate. While error introduced by lab protocols is hard to eliminate completely, it can be minimized and identified with proper procedures. Specifically, there are a number of pre-existing software packages focused on batch effects, including VOOM, SNM, ComBat-seq, and others (32–35). Relatedly, other confounding influences may not be known or measured but nevertheless create false associations or hide true associations between predictors and response variables. The most common examples of unknown confounders in microbiome studies are unmeasured environmental variables. The main issue with this specific type of confounder is that the model can detect and use these associations to form predictions without the user being aware, potentially causing the model to perform poorly when the confounder is different or absent. Exploratory data analysis (EDA) is a critical first step for identifying known or unknown confounders, as is employing robust external test data, and assessing model residuals (see below).
“Garbage in/garbage out (GIGO)” (data cleaning)	Samples and features with low read counts should be treated with caution. Additionally, low biomass samples have a high risk of contamination, and spurious taxa can, therefore, be identified by models as being “important” when they are not truly functional (or even present) in the experimental system. Typical “best practices” in data cleaning include performing abundance and prevalence filtering to remove rare or spurious taxa (36, 37). Additionally, adding “blank” samples at all steps of data generation and processing (sample collection, DNA extraction, PCR, sequencing) to identify and remove potential contaminants is important for all microbiome studies and especially for machine learning modeling where models can detect and amplify the importance of rare taxa (37)

*“Exploratory data analysis is an attitude, a flexibility, and a reliance on display, NOT a bundle of techniques.”*

*–John Tukey*

EDA is a critical first step in any analysis to gain familiarity with the data set. At this stage of data analysis, one should visualize the distribution of a data set, take key statistical metrics to identify outliers, and look for potential confounders. Doing so can help inform how to process the data prior to modeling, identify which ML model is most appropriate, and determine whether ML is needed at all. For example, if a histogram of the distribution between observed classes (e.g., number of samples per category) shows the data set to be highly unbalanced, one may decide to resample the data, avoid evaluation metrics such as accuracy, select a model type that performs well on unbalanced data (e.g., XGBoost [38]), or build a model that penalizes incorrect classifications of rare classes. If a large number of outliers are observed, and those outliers are likely to be spurious, one can either eliminate outliers or change the model performance metric for optimization (see Step 7 below). If the data are sparse and the distribution of variables has a “long tailed” distribution (i.e., few taxa or genes are common and most are rare, a common feature of microbiome data), one can consider using a feature selection process (see Step 4 below). If a batch effect is observed (39), one may need to take batch information into account prior to building models, especially when splitting data into testing and training sets (explained below). There are many other ways to examine data visually and statistically that will give any model a solid foundation, including identifying confounding variables, re-expression of data through transformations to better visualize data, and more. EDA is not a list of techniques that must be done before model building, rather it is an interactive exercise between a researcher and their data to find relationships and salient features. For more information on EDA, see references 26, 40–42.

Another pre-modeling step to consider with microbial ecology data is the level of taxonomic or genetic resolution to use as the input for the model. Amplicon sequence variants (ASVs) or operational taxonomic units (OTUs) are likely sparsely distributed across samples and, thus, could contribute noise but may be the resolution needed to identify associations to the response variable. For example, many plant pathogens are closely related to plant beneficial microorganisms (e.g., *Pseudomonas* species [43, 44]), so broad levels of taxonomic categorization may not be useful when trying to predict plant-microbe interaction outcomes. However, other patterns of interest could emerge at broader taxonomic classifications, and the appropriate level of resolution will depend on the questions being asked. Similarly, one can choose whether to train a model on individual genes or gene variants versus broader functional gene categories depending on the experimental question and the distribution of the data set at various levels of genetic resolution. Broader taxonomic categories (genus, class) or metabolic pathways may be appropriate predictors depending on the questions being asked and could result in less biased, more broadly applicable models (at the trade-off of reported internal performance) (29).

Finally, in addition to the data considerations listed in Table 2, the bioinformatic algorithms used to process raw sequence data can introduce errors and biases that, while unavoidable, are important to consider and communicate. For example, the choice of algorithms used to cluster 16S rRNA gene sequences based on the percentage of sequence similarity can result in very different input data tables. Similarly for metagenomic data, the binning methods or alignment algorithms employed can drastically change the genes and genomes identified and used in the models. These challenges and associated suggestions are covered elsewhere (45, 46) and apply regardless of the whether statistics or machine learning approaches are used. However, we emphasize that it is critical to include all details of bioinformatic processing steps (including software version numbers and settings) when reporting results, so readers understand the context with which the machine learning models were developed.

### Step 3: Split the data into a training and test set (with consideration of study biases)

Something that is critical to do when employing machine learning models that is not typically done in traditional statistical modeling is splitting up the data set into separate subsets: a “training” set, a “testing” set, and, in some cases, a “validation” set. The idea is to develop the model on a portion of the data (“training” and “validation”) and then evaluate the final model performance on the held-out portion of the data (“testing”). The validation set is typically used within the training period and serves as an internal test set for iteratively training and improving models, before the true test set is used to evaluate the final model, but not all studies have enough samples for this three-way split. Testing the final model at the end of the model development process enables the calculation of model performance on “novel” data the model has not encountered and is a good way to identify potential overfitting (see Table 1). Typically, a majority of the data is used in the training set (70:30, 80:20, 70:20:10), but splitting already small sample sizes common in microbiome studies (*n* = 10s-100s) can result in noisy models due to low volume of input data. One technique to overcome the shortfalls of the train-test split methodology is k-fold cross validation (see Table 1) (47). Although this technique can be useful when applied to small data sets, it comes at the cost of high variance (less likely to apply to external data sets; see Table 1) and is computationally expensive.

**Fig 1:**
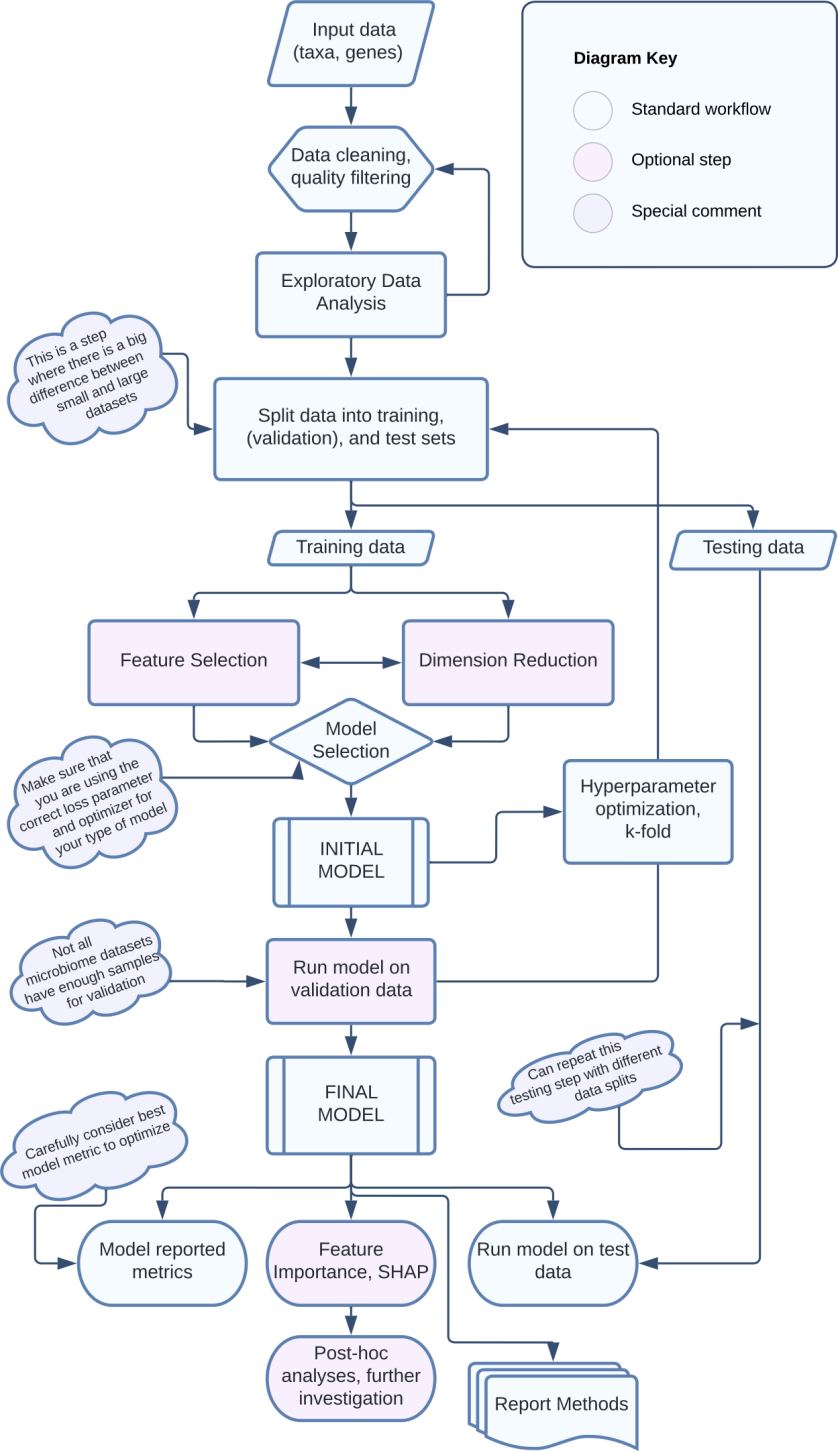
A schematic diagram of the steps for developing and applying supervised machine learning models.

While it is common to simply randomly assign samples into training and test sets, doing so without considering specifics of the data set can lead to poor (or overestimated) model performance. Biases in microbiome studies are common and must be explicitly addressed in model construction and transparently communicated when reporting results. Whalen et al. offer a discussion of how biological data sets can break assumptions of machine learning models: namely, samples across training and test sets should be, but often are not, independent with identical distribution patterns (48). For example, unbalanced classes where there are very few samples in one category (e.g., samples collected from diseased hosts) can lead to inaccurate models. Some options to mitigate unbalanced classes are over-sampling the minority class or under-sampling the majority class (48). Another common risk in microbiome data sets is the potential for information to “leak” from the training data to the test data, resulting in inflated model performance estimates. Specifically, having biological or technical replicates, or samples from the same study or cohort, that span both training and testing sets can lead to artificially high model performance because the test data is not truly “unseen” by the model. “Block splitting” keeps such related samples together in a split, minimizing this sharing of information with the test set (9, 49). Finally, any transformation of data that uses information across samples (scaling, standardization, principal components analyses), or feature selection (see below), should be applied separately to training and test data sets after being split for similar reasons (Fig. 1) (48, 50).

### Step 4: Perform feature selection

After determining that a machine learning approach is appropriate for the data and question, and after inspecting, cleaning, and splitting the data appropriately, a next step that is often helpful when working with microbiome data sets is to reduce the size of the input data (known as “feature selection”). Even after removing rare or spurious taxa or genes in the data cleaning step, including all remaining predictors in a typical microbiome data set can still result in overfitting, i.e., there is a higher chance that taxa or genes could be used in the model due to random chance (related to the “multiple testing problem” [51]). When a model has too many parameters, it can consider combinations of parameters that are so specific to the small set of samples that the result is more like a fingerprint than a heuristic—an example of extreme overfitting (52). For example, when trying to predict the environmental preferences of members of a specific taxon (53), a complicated model might identify the exact environmental parameters for every single strain input as training data with very little generalizability to other members of the broader taxonomic group. On the other hand, a model with fewer input strains would likely perform more poorly on the training data but could be more generalizable to other members of the taxon.

To help mitigate these issues, it is possible to eliminate the “noisy” predictors that are not likely related to the response variable of interest. For example, there are often hundreds if not thousands of bacterial taxa in a single microbiome sample, and many of these taxa may either be rare (30), dormant (54), or not relevant for the process being modeled. There are many software packages that will automate the feature selection step (e.g., permutation analyses, Boruta, Wrapper, Relief [55–57]), and many follow similar methods of iteratively shuffling values in the data set to determine which features perform better than would be expected if the values were random. After automated feature selection, it is important to inspect the chosen features prior to modeling to make sure that they are (biologically) reasonable. A check of whether the taxa or genes identified could conceivably be relevant to the response value or experimental system is a common-sense step that is essential and should not be overlooked.

Another option is to perform a more manual feature selection, where features are selected based on biological knowledge or model performance observation. For example, when predicting postmortem intervals based on microbiome data from different sources, information on skin and soil microbial taxa were found to be best for prediction, so samples from other sites (e.g., abdominal samples) were eliminated from the final model (58). Note that it is important to perform feature selection after data are split into training and test sets as performing feature selection on the entire data set could introduce biases into the final model, making the model performance appear better than it is and the model less likely to be applicable to other external data sets or situations (50).

### Step 5: Choose a model

Across the many different types of ML models available, how does one select the “best” model for a given situation? Note that ML models can be used for both classification and regression problems. Classification models predict discrete factors or categories in response variables, and regression models are used for predicting continuous response variables. For example, identifying taxa associated with diseased versus healthy individuals is a classification problem with categorical response data. In contrast, identifying genes predictive of bacterial pH preferences is a regression problem with continuous response data (3). The structure of the input data (as detailed in the above sections), the nature of the response data (e.g., categorical or continuous), and the goal of the modeling effort all help determine which model is likely to work best. Random forest (RF) (59) is one of the most commonly used modeling methods in microbial ecology. RFs are often leading performers in side-by-side model comparisons (13, 60, 61) and are known to be resilient to overfitting and less dependent on scaling or normalization procedures. While random forest models can be used in both classification and regression problems, they are more commonly used for classification because RF cannot predict values outside of training data in regression problems. Random forest models are an example of ensemble learning (many simple models combined together). Gradient boosting models (e.g., XGboost [38]; see Table 3) also fall into the category of ensemble learning. See Table 3 for brief descriptions of several other ML models often used for microbial ecology problems. For more details on these and other machine learning models, and for performance comparisons across multiple model types, see Hernández Medina et al., Naloufi et al., Song et al., and Zhou and Gallins (10, 13, 62, 63). Finally, we note that there are numerous software options available for testing and selecting machine learning models, many of which are implemented in Python and R. These tools are constantly changing and updating, and we, thus, refrain from making specific software recommendations here. Of note, recently automated ML (AutoML) has become an attractive option to new users as these systems are designed to choose an appropriate algorithm for use. However, beginners using AutoML tools need to be cautious of using the tools without understanding the underpinnings and biases of the models generated.

**TABLE 3:** Examples of machine learning models used in microbial ecology

Model name	Purpose/description	Use case/limitations
Support vector machines	Segregate the data into two groups on either side of a linear boundary, maximizing the distance between them. Despite the linear nature of SVMs, they can be used on data that is not linearly-separable	SVMs need to be scaled to prevent outliers from unduly impacting the distance metric and tend to perform poorly on noisy data and big data.
Naïve Bayes classifiers	Classifiers that use posterior probabilities based on Bayes theorem	Fast even on high-dimensional data, and less sensitive to scaling. Assumes independence of features.
Linear/logistic regression	Supervised probabilistic model for binary classification	Easy to interpret and simple to implement but often perform poorly with non-linear classification and non-normal data.
Random forest	An ensemble method that relies on the majority consensus of resampled decision trees	Resilient to overfitting, less dependent on scaling or normalization procedures, cannot predict values outside of training data in regression problems
XGboost	An ensemble of decision trees that are optimized for minimization of the loss function using gradient descent	More reliable than random forests for unbalanced classes and preferable to random forests when the aim is to decrease bias

### Step 6: Choose tuning parameters (hyperparameter optimization)

After identifying the type of machine learning model to use, it is important to test and tweak the settings that determine how that model learns, called “hyperparameters,” to optimize model performance and limit overfitting. Hyperparameters are custom to the model type used and can include the number of decision trees in a random forest or the number of features (e.g., taxa or genes) used for making decisions at each node in a decision tree. There are several methods one can employ to identify the optimal hyperparameters. Grid search examines every possible combination of hyperparameters, eventually presenting the combination that results in the best model performance. However, this exhaustive methodology makes it extremely computationally intensive. As a less intensive and time-consuming alternative, a set number of hyperparameter combinations can be selected randomly although this may not result in the theoretical best possible model. Finally, Bayesian optimization uses previous optimization results to select new hyperparameter combinations. Bayesian hyperparameter optimization typically results in the selection of high-quality hyperparameters in a relatively short amount of time and is increasingly becoming a method of choice (64). While many ML packages and programs have default settings that enable models to run without hyper-parameterization, skipping this step often results in models with lower performance (65).

### Step 7: Evaluate model performance

Once a model has been constructed, performance of the model should be evaluated using both metrics reported by the model and testing on withheld and/or external test data. Which metrics are best for assessing model performance depends on the type of model, the type of response variable (categorical or regression), and also the specific use-case scenario. Table 4 has details to help determine the appropriate metrics. Briefly, “model-reported” metrics for classification models include accuracy, precision, recall/sensitivity F1, Kappa, and (AU)ROC/(AU)PR curves (see Tables 1 and 4). The most important consideration when choosing metrics for classification models is which type of misclassification has the highest cost, or which type of prediction is most important (see Table 4) (66). For regression models, R^2^, (R)MSE, MAE, and (S)MAPE are all model-reported or internally calculated metrics that can be used for evaluation (67). Note that RMSE, one of the more commonly reported metrics, is measured in the same units as the response variable, making it inherently easier to interpret and compare across models.

**TABLE 4:** Model evaluation metrics

Model type	Model metrics	Use case/description	Example
Classification binary: two categories OR multi-class: three+ categories	AUROC curve	A metric measuring a classifier’s ability to distinguish between positive and negative classes. More valuable as a metric when classes are balanced and the cost of misclassification is consistent between classes.	Diagnostic prediction for diseases (e.g., identifying multiple diseases with fecal microbiome data [68]).
	AUPR curve	A metric measuring the tradeoff between precision and recall for a binary classifier. Superior to (AU)ROC when classes are not balanced. Optimizes for true positives.	Diagnostic prediction for rare diseases (e.g., predicting liver disease risk with gut microbiome data [69])
	F1	The harmonic mean of precision and recall for a binary classifier. Optimize for F1 score when seeking a balance between precision and recall.	Diagnostic prediction for diseases (e.g., diagnosing IBS from gut microbiome data [70]).
	log-loss	Measures the probability that predicted outcomes for a classification problem match observed outcomes. Optimizes for minimizing false negatives.	Discriminating between multiple different diseases (71). Good for multi-class comparisons, sensitive to unbalanced classes (14).
	Kappa	Takes into account the probability that correct classification can be achieved by random guessing, making it a more valuable metric than accuracy and F1 score for both unbalanced and multiclass data.	Predicting categorical soil health metrics (72).
	Accuracy	The ratio of correctly categorized instances over the total number of instances; best used when classes are balanced and the costs of both false positives and false negatives are symmetrical.	Predicting the health status of sea sponges (73).
Regression	*R* ^2^	The coefficient of determination—how much of the variance in the response variable is explained by the predictive variable(s).	Predicting continuous variables such as dissolved organic carbon (60) and contamination metrics in rivers (74).
	(R)MSE	“(Root) mean square error.” Metric measuring the Euclidean distance between observed and predicted values for a regression problem, larger errors are penalized more heavily than smaller ones. Lower values are better. “R” is the square root of MSE and is reflective of the units of the response variable, making it a good metric for cross-model comparisons.	Comparing model effectiveness for predicting *E. coli* presence in manure (75).
	MAE	“Mean absolute error.” Metric measuring the magnitude of errors between observed and predicted values for a regression problem. Does not give weight to larger errors as with MSE, protecting outliers. Lower values are better.	Predicting post mortem interval (PMI) using soil and skin microbiomes (58).
	MAPE	“Mean absolute percentage error.” Metric measuring the percentage difference between observed and predicted values for a regression problem, which can help with interpretability. However, it can be negatively affected by sparse data and outliers making it a poor choice for many applications in microbial ecology. SMAPE is the symmetric mean absolute percentage error and is bounded between 0% and 200%, lower being better.	Predicting water quality and contamination (62).

For regression models, assessing model residuals (the difference between predicted and true values) can reveal whether the model is missing important information (76). Residuals should be independent and normally distributed, indicating that any error in the model is random. If residuals are not normally distributed, checking whether they correlate with experimental variables like batch or geographic location can help identify whether confounders are affecting model performance.

It can be hard to determine whether a model is overfit (good performance on training data but poor prediction on test data) by evaluating the model-reported metrics alone because the models are built to optimize those metrics. Running the final model on a withheld portion of the data, or an entirely separate data set, will help identify potential overfitting and provide a better indication of true model performance. As an impressive example of model validation using multiple external test sets, Yuan et al. developed a model to predict soil disease status using bacterial and fungal soil information and tested the model on 19 external publicly available data sets as well as on 20 newly collected soil samples, demonstrating robust model performance across different data sources (61)

Since it is possible to have “lucky” (or “unlucky”) splits of the data into training and test sets, such that by chance the test data are more or less easily predicted, it is recommended to add confidence intervals to both model metrics and performance on test data. This is done by repeating the train-test split multiple times with different random seed values (a version of cross-fold validation) and combining performance and predictive metrics together for an average performance value with 95% confidence intervals (15).

Finally, another option for evaluating machine learning model performance is to compare model results to the same models with shuffled data (shuffling either feature data or response labels). For example, in an assessment of the predictive capacity of fungal and bacterial information for the classification of human tumors, extensive “control” models were developed with shuffled data to show that models with true input were detecting meaningful biological signals (77, 78).

### Step 8: Interpret results of model: what does it mean?

Often the goal of applying ML models in microbial ecology is not just to produce a good model *per se*, but rather to infer some biologically meaningful information by identifying and learning about the specific features that go into that model. While machine learning models are generally more predictive at the cost of being less interpretable, several models and post-modeling analyses can provide additional information about how specific features impact model decisions. For example, random forest models provide feature importance rankings, which list how important each feature is in the model (as determined by loss of accuracy when a feature is removed). By using importance rankings from random forest models, Smith et al. (2) showed that the taxa most important for predicting environmental contamination were known to interact with the main contaminants (nitrate and uranium), further validating the model. Permutation analysis offers a method for generating feature importance rankings for nonlinear models if not already provided by the model (15, 79).

Shapley additive explanations (SHAP) analysis is another convenient tool for understanding and interpreting the outputs of ML models. SHAP analysis can be used to determine the weight and direction of each feature in the model output and can also help a researcher understand which factors contributed the most to any individual prediction (80). For example, SHAP was used to identify the relative importance and impact of specific genes for predicting the pH preferences of bacteria (3).

Those features (genes or taxa) identified as being important in the model can be the targets of additional *post hoc* analyses or further hypothesis testing. Perhaps most critically, it is important to carefully examine the taxa or genes identified by the model as being important to see whether they make sense. For example, a paper demonstrating the predictive capacity of tumor-associated microbiomes was called into question when an archaeal genus known only to occur in hydrothermal vents (*Ignicoccus*) was reported as important for predicting prostate cancer (29, 77). Additional analyses that investigate independent relationships between important predictive features and the response variable can yield more biological information than the model alone can offer, potentially identifying promising avenues for future research. As just one example, Wilhem et al. identified the bacterial taxa most important for predicting soil health metrics and found that ammonia oxidizing bacteria were often predictive of low-quality soils, perhaps due to consistent fertilizer use (72).

### Step 9: Report detailed methods of model generation and interpretation

As with any experimental endeavor, it is important to report detailed information on how models are selected, generated, and evaluated. This includes communicating information about how raw data were cleaned (including reporting information on “contaminants” [36]) and how data were normalized, transformed, or otherwise processed before being used as input into the models. Additionally, reporting exactly when and how test data were separated from training data, and any variation in cross-fold performance should be reported. A brief explanation of which metrics were chosen for model optimization and why also helps with downstream assessment and reproducibility (15). See Table 5 for a list of specific information that is helpful to report when describing ML modeling methods.

**TABLE 5:** Information that is useful to report when describing ML modeling efforts

Raw data processing	Contaminant removal, abundance/prevalence filtering, normalization, transformation. See references 15, 36.
Data splitting	Ratios of train, test, validation split; performance variation with different data splits; source of external test sets
Feature selection	Method of feature selection, when in process feature selection was applied, whether cross-folded
Model type	Type(s) of model(s) used and why, metrics used for model evaluation and optimization
Hyperparameters	Final hyperparameters selected for model, and methods used for hyperparameter optimization
Code	Open-source code and publicly available data are ideal but not always required

Finally, very rarely are samples collected for any study perfectly representative of the broader population (e.g., microbiome samples in a medical study conducted at a single hospital, or soils collected from historically managed experimental fields). While this is often inescapable, transparently communicating what biases were considered during model development is crucial when reporting machine learning results. Biases of the overall sample collection and study design that are not addressed within the model should be clearly communicated with accompanying disclaimers for interpreting the results. For example, findings from a study on the gut microbiomes of predominantly young, white men will likely not extrapolate to a more diverse population. While these challenges are not unique to machine learning, given the application of prediction in ML, it is important for authors to emphasize the limitations of when the model predictions may fail (e.g., in an environment distinct from the study environment, or in human or animal populations that differ from study cohort).

### Conclusions

By offering a consolidated framework for deciding when and how to employ ML models in microbial ecology, we hope to assist with application of this methodology in the field. Note that the authors of this paper do not claim expert domain knowledge in the mathematical underpinnings of machine learning models, as is the case for most microbial ecologists using ML methods. We also recognize that the tools and recommendations for ML methods are changing rapidly, as is the scope and size of molecular microbiome data. The increasing size of microbiome data sets and increasing familiarity with ML tools is resulting in creative and exciting applications, such as improving metagenomic and protein annotations (81), revealing complex inter-kingdom interactions with multiple data types (82), or identifying required or ideal growth parameters for culturing (83). Machine learning is proving to be a useful and increasingly common method of analysis across biological disciplines and can be used effectively when done correctly and communicated clearly. However, like all statistical approaches, it is important to understand how and why to apply ML models and carefully consider their limitations when interpreting their output.
